# *In vivo* approach on femur bone regeneration of white rat (*Rattus norvegicus*) with the use of hydroxyapatite from cuttlefish bone (*Sepia* spp.) as bone filler

**DOI:** 10.14202/vetworld.2019.809-816

**Published:** 2019-06-14

**Authors:** Aminatun Aminatun, Fadhilah D. E. Handayani, Prihartini Widiyanti, Dwi Winarni, Siswanto Siswanto

**Affiliations:** 1 Department of Physics, Faculty of Science and Technology, Universitas Airlangga, Surabaya, East Java 60115, Indonesia; 2 Program Study of Biomedical Engineering, Department of Physics, Faculty of Science and Technology, Universitas Airlangga, Surbaya, East Java 60115, Indonesia; 3 Department of Biology, Faculty of Science and Technology, Universitas Airlangga, Surabaya, East Java 60115, Indonesia

**Keywords:** anatomical pathology, bone filler, cuttlefish bone (*Sepia* spp.), hydrothermal, hydroxyapatite

## Abstract

**Background:**

Hydroxyapatite (HA) from bovine bone has been widely used as bone filler in many fractures cases. HA can also be made from cuttlefish bone (*Sepia* spp.) that has abundant availability in Indonesia and contains 84% CaCO_3_, which is a basic ingredient of HA. However, research on the effects of HA from cuttlefish bone on bone regeneration parameters has not been done yet.

**Aim:**

This study aimed to determine femur bone regeneration of white rats (*Rattus norvegicus*) through the use of HA from cuttlefish bone (*Sepia* spp.) as bone filler.

**Materials and Methods:**

HA was made using the hydrothermal method by mixing 1M aragonite (CaCO_3_) from cuttlefish bone and 0.6 M NH_4_H_2_PO_4_ at 200°C for 12 h followed by sintering at 900°C for 1 h. *In vivo* test was carried out in three groups, including control group, bovine bone-derived HA group, and cuttlefish bone-derived HA group. The generation of femur bone was observed through the number of osteoblasts, osteoclasts, woven bone, lamellar bone, havers system, and repair bone through anatomical pathology test for 28 days and 56 days.

**Results:**

Anatomical pathology test results are showed that administration of bovine bone-derived HA and cuttlefish bone-derived HA increased the number of osteoblasts, osteoclasts, woven bone, lamellar bone, havers system, and bone repair at recuperation of 56 days. Statistical test using Statistical Package for the Social Sciences with Kruskal–Wallis and Mann–Whitney U-test was resulted in significant differences between the bovine bone-derived HA control group and the cuttlefish-derived HA control group. There was no significant difference toward the indication of bone formation through the growth of osteoblasts, osteoclasts, woven bone, lamellar bone, havers system, and bone repair in the bovine bone-derived HA and cuttlefish bone-derived HA groups.

**Conclusion:**

It can be concluded that cuttlefish bone-derived HA has the potential as bone filler based on the characteristics of bone regeneration through *in vivo* test.

## Introduction

Fractures are commonly caused by injury, accident, mechanical impact, or osteoporosis [1], which then result in bone regeneration. One method in handling functional damages and that can accelerate the process of bone regeneration is biomaterial implantation. This type of method that has been widely used is through bone substitution. Basically, the use of implanted bone substitution must be biocompatible to the surrounding tissues, non-toxic, and bioactive [2]. One of the natural ingredients that have been developed as the implant is hydroxyapatite (HA). HA, with the chemical formula of Ca_10_(P0_4_)_6_(OH)_2_, is one of the inorganic compounds that make up the hard tissue in the human body such as bone and stimulate the presence of osteoconductive which make HA suitable as a bone substitute [3,4]. HA is often used as bone implant material because it is bioactive, osteoconductive, non-toxic; also, it can create close bonds to bone tissue, supports cell proliferation processes, and does not cause inflammatory reactions [5,6].

The basic ingredients for HA consist of eggshells, coral, bovine bone, and cuttlefish bone [7-9]. Cuttlefish bone (*Sepia* spp.) is a type of seafood that is often used to feed birds and turtles. In addition, cuttlefish bone is relatively inexpensive, abundant in waters and contains 84% CaCO_3_. It has the potential to be used as a base material for HA, which meets the requirements as bone substitution material [10]. Research on the synthesis of HA with a source of CaCO_3_ from cuttlefish bone using hydrothermal method has been carried out by Siswanto *et al*. [11]. Based on this study, there was optimization of the hydrothermal method at 200°C for 12 h, i.e. the production of HA with high purity, high crystallinity, controlled size and morphology, and high reactivity [12]. Following this, the optimization of the sintering method has been carried out by Aminatun *et al*. [1]. The sintering method was carried out at 900°C for 1 h. High-Temperature heating can produce crystalline structure and crystallinity that are suitable for HA commercial. In the study, the compressive strength reached 11.79900±0.00057 MPa, which is fit for cancellous bone applications. Cytotoxicity test by MTT Assay on fibroblasts showed that HA from cuttlefish bone was not toxic with the highest cell viability of 90.89% [1]. The results of *in vitro* test showed that HA from cuttlefish bone is suitable for bone filler. The development of these results was carried out by *in vivo* test to determine the regeneration process of bone tissue.

Ideal bone filler is a material that can form strong bonds with tissues and stimulate new bone cell growth. This study is necessary to be conducted to produce HA, which can be a suitable material for bone implant for fractures. Bone filler from cuttlefish bone-derived HA was implanted in the femur bone of white rats (*Rattus norvegicus*). Cuttlefish bone-derived HA is expected to accelerate osteoblast cell growth and stimulate new bone formation. White rats (*R. norvegicus*) were chosen because the treatment tends to be easier, and they have bone structures similar to humans.

This study aimed to examine the effect of cuttlefish bone-derived HA on the growths of osteoblast, osteoclast, woven bone, lamellar bone, havers system, and bone repair.

## Materials and Methods

### Ethical approval

This study was approved by the Ethical Committee of the Veterinary Faculty Universitas Airlangga, Indonesia with the reference number 2.KE.075.05.2018.

### Materials

The materials used for sample making were cuttlefish bone (*Sepia* spp.), ammonium dihydrogen phosphate (NH_4_H_2_PO_4_), methanol p.a., distilled water, and HA from bovine bone. The materials used in the *in vivo* test were female white rats (*R. norvegicus*), Pehacain injection (anesthesia), povidone-iodine, ketamine, sewing thread, and gauze dressing for wound. The ingredients used for anatomical pathology preparation include nitric acid, formalin, chlorophyll, alcohol, xylol, distilled water, liquid paraffin, Mayer dye paints, Hematoxylin, Harris, comparative coloring paints of eosin, and Canada balm. Finally, the tools used in the research include tools for sample making and sample testing. Testing tools were PANalytical X’Pert PRO X-ray diffractometer and a set of tools for *in vivo* test.

### Synthesis of HA through hydrothermal and sintering methods

Synthesis of cuttlefish bone-derived HA in the present study refers to the synthesis that has been done by Aminatun *et al*. [1] and Siswanto *et al*. [11]. 1M CaCO_3_ and 0.6 M NH_4_H_2_PO_4_ solutions were mixed using a magnetic stirrer for 30 min. The CaCO_3_ was obtained from the lamellae part of cuttlefish bone which was made into powder using mortar; then, it was heated at 350°C for 3 h with an increase of 10°C for every heating. Afterward, the mixture of CaCO_3_ and NH_4_H_2_PO_4_ was transferred to a reactor which was then put into an electric oven to be heated at an optimal temperature of 200°C for 12 h. After the heating process, the sample was cooled down at room temperature and washed with distilled water using a magnetic stirrer until the pH was neutral. The last washing used methanol to limit the agglomeration of HA particles during the drying process. Then, the sample was filtered with filter paper and dried in an electric oven at 50°C until it was dry. The dried sample was then sintered at an optimum temperature of 900°C for 1 h to get maximum results in the production of HA. The results obtained from bone synthesis X-ray diffraction (XRD) characterization to ensure the content of HA and sterilization using Gamma.

### XRD test

XRD test used PANalytical X’Pert PRO X-ray diffractometer. The results obtained were the identification of the content composition of cuttlefish bone HA. In addition, the composition of Ca/P contained in HA Ca_10_(PO_4_)_6_(OH)_2_ could be seen, and no other compounds other than Ca/P from HA were formed.

### *In vivo* test

This study used experimental animals of 30 white rats (*R. norvegicus*), aged 3-4 months and weighed 200-300 g. The adaptation was done to the rats for 1 week. During the adaptation period, they were given standard food and drink. The adapted white rats were then anesthetized with ketamine in the area that was to be injured. Meanwhile, the use of a combination between 0.25 mg/kg medetomidine MED and 100 mg/kg ketamine was more suitable for general anesthesia [13]. The fracture was made to the femur of the rats with the direction of lengthwise incision in the femur bone; then, the muscle was separated from the bone with a raspatory and drilling of bone to make a defect with a diameter of 1 mm. The cuttlefish bone-derived HA and bovine bone-derived HA powder (0.5 mg each) were inserted into the injured femur bone. Afterward, the flaps were returned and stitched with silk 3/0 thread, then given povidone-iodine to fasten the healing process. The recovery was taken 2 h after surgery, and the rats were placed in a cage, they were fed and given drink. Furthermore, after the surgery, the rats were given 22 mg/kg bid of cephalosporin antibiotics for 2 weeks, 2 mg/kg bid of painkiller Rimadyl for 5 days and as to stop bleeding using transaminase and multivitamin 1×1 for 7 days, eye drop Levofloxacin 6×1 drops for 7 days than 4×1 drops for the next 7 days, and also lubricant (Regefluid^®^) for 1 month. The suture removal of the tarsorrhaphy was done 7-10 days post-operation. The control was done every 7 days to 2 times, then 1 month after [14]. Wound contraction is the key to wound healing, which contributes to minimization of infection and promotes rapid wound closure. Diclofenac and flunixin cause significant delays in wound contractions on day 4, day 7, and day 11. However, treatment with metamizole results in a slight decrease in contraction when compared with control [15]. The rats were taken care for 28 days and 56 days, because histologically bone regeneration was begun to occur.

### Analysis of anatomical pathology and statistics data

Anatomical pathology test of femur bone tissue was conducted by observing the femur bone tissue directly under a light microscope (Nikon H600L equipped with 300 megapixel DS Fi2 camera and image management software Nikon Image System from Japan Optical Industries Co., Ltd.) at 1000× and five fields of view. Observation of osteoblast cells was characterized by a round nucleus in the basal; a large osteoclast that contains nucleoli, woven bone with the characteristics of irregularly shaped matrices and regularly shaped lamellar bone, and havers systems forming nerve fibers containing connective tissue. The results of anatomical pathology test were analyzed by Statistical Package for the Social Sciences (SPSS) program using the two-way ANOVA parametric analysis method and non-parametric statistics. Non-parametric statistics were used to compare the distribution of samples with normal distribution using the Kolmogorov–Smirnov test. Different tests between two samples from two different populations were carried out through Kruskal–Wallis and Mann-Whitney U-test.

## Results

### XRD test results

**Figure-1:**
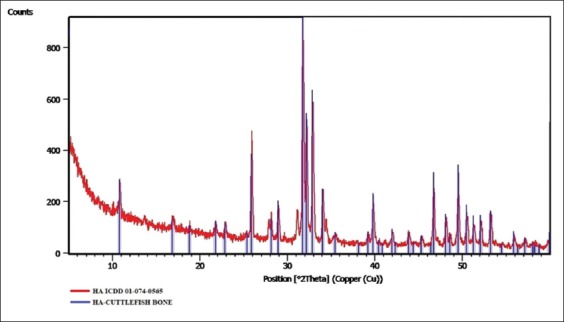
X-ray diffraction test results of hydroxyapatite from cuttlefish bone (*Sepia* spp.).

Results of XRD analysis showed that the content of HA sample synthesis from cuttlefish bone (*Sepia* spp.) is 100% HA (Ca_10_(PO_4_)_6_(OH)_2_), as shown in Figure-1.

The XRD spectrum in Figure-1 shows compliance with ICDD 01-074-0565. The results obtained from XRD test showed that no compound other than HA was formed.

### Anatomical pathology test results

Anatomical pathology test results were obtained from observing microscopic slides of femur bone tissue of the experimental animals. Each treatment group used three experimental animals with the treatment times of 28 and 56 days. From the slides results, anatomical pathology test results can be analyzed by observing the degree of healing in bone fractures after being treated.

### Anatomical pathology results of the treatment groups for 28 days

**Figure-2:**
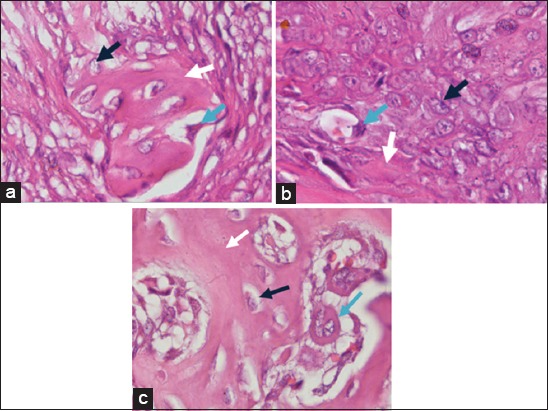
Observation results of osteoblasts and osteoclasts on day-28 with (a) control treatment group, (b) bovine bone treatment group, and (c) cuttlefish bone treatment group. Black arrows show osteoblasts, white arrows show bone matrix formation, and blue arrows show osteoclasts (HE staining, 1000×).

Observation results of osteoblasts, osteoclasts, woven bone, havers system, and bone repair of each treatment were obtained. Figure-2 depicts the results of anatomical pathology tests for observation of osteoblasts and osteoclasts for 28 days of treatment.

**Figure-3:**
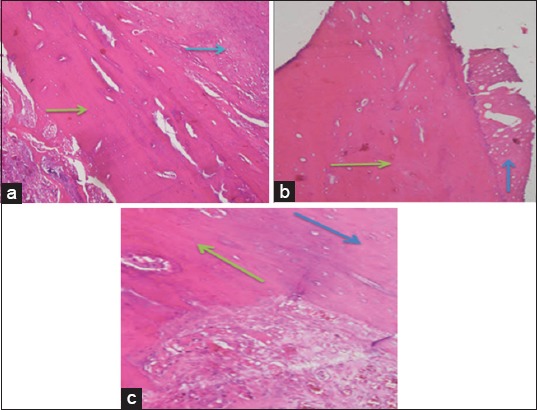
Observation results on day-28 with (a) control treatment group, (b) bovine bone treatment group, and (c) cuttlefish bone treatment group for woven bone and lamellar bone. Blue arrows show woven bone, green arrows show the formation of lamellar bone that forms the havers system (HE staining, 1000×).

In Figure-2, osteoblast was characterized by the presence of a rounded nucleus in the basal cell that contains one to three nucleoli. Osteoclast was characterized by large cell and there are many nuclei. Meanwhile, the observation of woven bone to lamellar bone was characterized by differences in shape and structure. In the woven bone, the resulting matrix was irregular while in the lamellar bone, it was irregularly. The havers system was formed from a set of lamellar bone that forms circular compact bone into a new bone (bone repair). The observation results of woven bone, lamellar bone, havers system, and bone repair are shown in Figure-3.

### Anatomical pathology results of the treatment groups for 56 days

**Figure-4:**
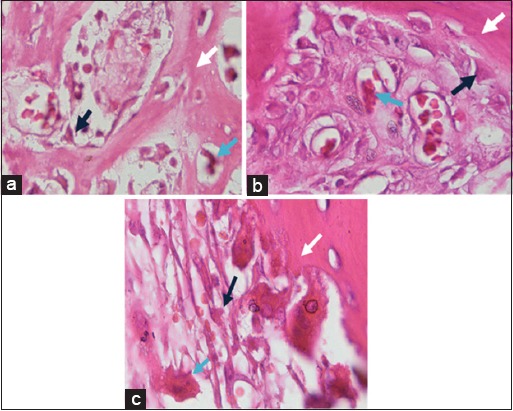
Observation results of osteoblasts and osteoclasts on day-56 with (a) control treatment group, (b) bovine bone treatment group, and (c) cuttlefish bone treatment group. Black arrows show osteoblasts cells, white arrows show matrix formation, and blue arrows show osteoclasts cells (HE staining, 1000×).

Observations were seen from five fields of view that was randomly obtained at the repetition of experimental animals. The observation results of osteoblasts and osteoclasts for 56 days of treatment are presented in Figure-4.

In 56 days of treatment (Figure-4), osteoblasts were more mature than those in 28 days of treatment (Figure-2). The number of osteoblasts in the bovine and cuttlefish treatment groups was much more than in the control treatment group. This indicates that the administration of bone filler from bovine bone and cuttlefish bone can interact with the subject’s bone. Osteoclasts were found in bones that were going through the remodeling process, both physiologically and pathologically such as tumors or bone fractures.

**Figure-5:**
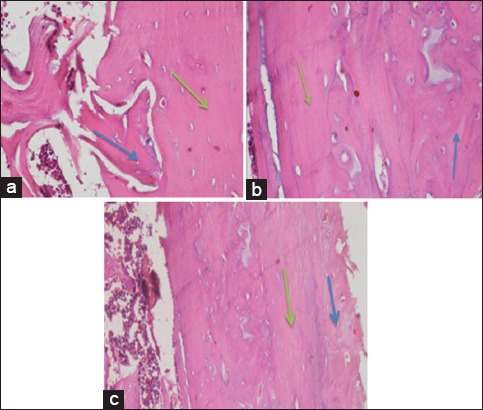
Observation results on day-56 with (a) control treatment group, (b) bovine bone treatment group, and (c) cuttlefish bone treatment group for woven bone and lamellar bone. Blue arrows show woven bone; green arrows show lamellar bone that forms havers system (HE staining, 1000×).

The following observations results of woven bone, lamellar bone, havers system, and bone repair at 56 days of treatment (Figure-5) were almost the same as the results at 28 days of treatment (Figure-3). The differences lie in the number of forming cells that were getting more mature and perfect new bone regeneration occurs. The remodeling process in the bone regeneration stage takes months to years for a complete regeneration process. Data from the number of cells produced were processed using SPSS to determine differences in each treatment group with recuperation time. Observation results of woven bone, lamellar bone, havers system, and bone repair at 56 days of recuperation time are shown in Figure-5.

### Results of data analysis based on histopathological observation for a treatment time of 28 days and 56 days

**Figure-6:**
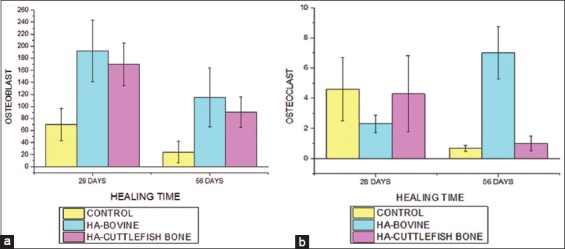
Graphs of mean differences from (a) number of osteoblasts (b) number of osteoclasts in the control group, bovine bone, and cuttlefish bone at 28 days and 56 days.

Data analysis based on histopathological observations for treatments of 28 and 56 days was done by observing the anatomical pathology test and the statistical test. The observation of anatomical pathology results resulted in data on the number of osteoblasts and osteoclasts, which is shown in Figure-6.

Observation of histopathological data results was continued by statistical tests of two-way classification analysis (Two-Way ANOVA). The results obtained show that the treatment group and recuperation time affect the number of osteoblasts and osteoclasts. In the number of osteoblasts, there is no difference between the types of HA and recuperation time. However, there is a difference between the types of HA and the recuperation time toward the number of osteoclasts.

**Figure-7:**
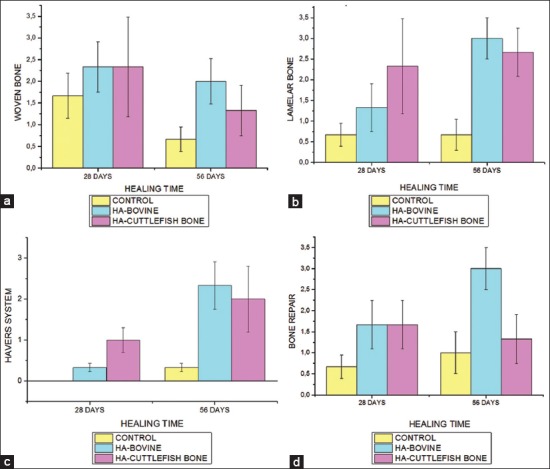
Graphs of mean differences from (a) number of woven bones (b) number of lamellar bone (c) numbers of havers system (d) number of bone repair in the control group, bovine bone and cuttlefish bone at 28 days and 56 days.

The statistical tests result for observations of woven bone, lamellar bone, havers system, and bone repair used nonparametric analysis, while osteoblasts and osteoclasts used parametric analysis. The following observations for treatment of 28 and 56 days are presented in Figure-7.

After the anatomical pathology test, statistical test was performed to determine the differences between each treatment. The statistical test for observing woven bone, lamellar bone, havers system, and bone repair used nonparametric, namely Kruskal–Wallis. The results obtained for woven bone (p=0.157>0.05), lamellar bone (p=0.015<0.05), havers system (p=0.055>0.05), and bone repair (p=0.016<0.05). It is known that there are significant differences in the lamellar bone and bone repair, so statistical analysis using Mann–Whitney was performed.

Comparisons on the statistical results of lamellar bone on each treatment are control treatment with bovine HA treatment (p=0.026<0.05) and control treatment with cuttlefish bone HA treatment (p=0.009<0.05), it shows a significant difference. However, there was no significant difference between the treatment of bovine bone-derived HA and the treatment of cuttlefish bone-derived HA (p=0.589>0.05). Based on the results, the treatment of bovine bone-derived HA with the treatment of cuttlefish bone-derived HA yields a not so different value.

Meanwhile, the results of bone repair on each group are the control group with bovine bone-derived HA treatment shows p=0.015<0.05, the control group with cuttlefish bone-derived HA treatment shows p=0.093>0.05, and the treatment group of bovine bone-derived HA with the treatment of cuttlefish bone-derived HA shows p=0.093>0.05. This shows that there was a significant difference between the control group and the bovine-derived HA treatment group, but there was no significant difference between the bovine bone-derived HA treatment group and the cuttlefish bone-derived HA treatment.

## Discussion

Anatomical pathology evaluation is the primary means in assessing the effect of bone substitutes in tissues. Based on the anatomical histopathology observation process of post-operative female white rats (*R. norvegicus*), there was a regeneration process due to the administration of bone filler from bovine bone and cuttlefish bone. Bone filler functions as filler for damaged bones, and generating new bone cell growth. It is biodegradable or resorbable, so they will disappear when new bone cells have grown [16]. Bone regeneration begins with the inflammatory stage. The inflammatory stage occurs for 1-3 days and disappears almost completely 1 week after the damage occurs which causes the formation of hematoma (localized collection of blood) whose end of the fragment is invaded by a macrophage [17]. After 5 days, the formation of fibrin, new blood vessels, and osteoblasts occurred due to the use of bone filler. Bone filler triggers the formation of osteoblasts. Osteoblasts play a role in synthesizing collagen and glycoprotein components. Osteoblasts also synthesize bone matrix elements which are mostly composed of type 1 collagen and glycoproteins. The osteoconductive and bioactive properties of HA accelerate the process of mineralization, which helps in forming new bone. Osteoblasts precipitate new organic matrix elements that form osteoid, namely uncalcified bone tissue [18]. Mineralization calcium phosphate is extracellularly stimulated by osteoblasts, which produce a collagen-rich matrix called osteoid [19]. HA directly bonds with the live bone after implantation in cases of bone defects. This feature increases appropriate vascularization and proliferation, and guides bone regeneration without causing any local or systemic toxicity [20].

The osteoblast cell membrane contains several enzymes, one of which is alkaline phosphatase, which plays a role in the process of bone calcification. When bone formation is almost complete, some of the osteoblasts are calcified into osteocytes [18]. Alkaline phosphatase enzymes cause an increase in phosphate concentration so that the formation of calcium phosphate bonds in the form of HA crystals will settle in the bone. When osteocytes detect damage in bone, osteoclasts begin to activate resorption of bone matrix and make the damage filled by osteoblasts [21]. Bone resorption causes the activity of osteoblasts and osteoclasts to increase again that continuously will form woven bone.

In the 28^th^ day of observation, woven bone was formed. The formation of woven bone was included in the repair stage. Woven bone is formed from accumulated minerals and increased activity of osteoblasts. Then, the formation of these activities forms the lamellar bone. Lamellar bone begins to harden and form woven bone between fractures fragments incorporated in fibrous cartilage (fibrocartilago) [22]. At 56 days of treatment, lamellar bone was formed perfectly. In the lamellar bone, collagen fibers are arranged parallel and circular in the center which results in the formation of harvers system. The havers system is formed from blood vessels and nerve fibers that are filled with binding tissue. Based on Shapiro’s research, the formation of havers system occurs at 7^th^-8^th^ weeks [23]. The havers system passes through the edges of bone repair that takes place in the matrix and blood vessels. Bone repair occurs in 8-12 weeks after a fracture occurs. Bone repair is included in the remodeling stage, which is the final stage of bone regeneration.

From the test results at a treatment time of 28 and 56 days, it was found that in the bovine bone treatment group, there was an increase of osteoblasts and osteoclasts compared with the control group. It also happens in the cuttlefish bone treatment group, which had an increase in osteoblasts and osteoclasts compared to the control group. Both of these increases occurred on day 56. This shows that the type of material used affects the process of bone formation. The longer the recuperation time, the closer the bone regeneration to perfection. Regeneration parameters can be seen from the presence of bone regeneration parameters such as: Woven bone, lamellar bone, havers system, and bone repair. In the comparison of regeneration parameters for the treatment group, there are significant differences in lamellar bone and bone repair. Significant differences occurred in the lamellar bone to the control group with bovine bone treatment and cuttlefish bone treatment. In this study, the process of bone formation is still being continued.

Remodeling was preceded by osteoclast resorption which caused lacuna resorption with various depth between 60 µm in adolescents and 40 µm in adults. The resorption period takes approximately 30-40 days and is followed by bone formation up to 150 days [24]. Bone repair is a remodeling stage or the final stage of the bone regeneration process. During the remodeling process, osteoblasts will fill the space between the woven bone and the trabecular bone. Then, osteoblasts replace trabecular bone into the compact bone. The HA content helps bones to form, induce, and produce new bone [20,25].

The success rate of the bone healing process is indicated by the formation of a new bone matrix that is secreted by osteoblasts, which will then be oscillated. HA with a rough surface will facilitate the infiltration of osteoblast cells. Attaching osteoblasts to the bone graft surface progresses slowly depending on the compatibility of the bone graft material surface [26]. Osteoblasts will deposit new bone and blood vessels will accompany the osteoblast. During the mineralization process, the ends of the bone fragments gradually become covered by increasing fusiform mass containing woven bone. The more minerals that are deposited, the harder the callus formed [27,28]. It is also proven that the treatment group and the recuperation time affect the bone regeneration process. Moreover, various recuperation times affect the mineral uptake, the immobilization of bone fracture, and the growth factor which is embedded in the graft. The longer the recuperation time, the more perfect the bone regeneration formed.

## Conclusion

HA from cuttlefish bones (*Sepia* spp.) affects the process of bone growth and the recuperation time. The optimum recuperation time in bone formation occurs on day 56. The longer the recuperation time, the more new bone will be formed, and the more perfect bone regeneration occurs. The statistical tests using SPSS with Kruskal–Wallis and Mann–Whitney U-test produced significant differences between the control group with bovine bone-derived HA and the control group with cuttlefish bone-derived HA. Whereas in the bovine bone-derived HA and cuttlefish bone-derived HA groups, there was no significant difference in the indication of bone formation through the growth of osteoblasts, osteoclasts, woven bone, lamellar bone, havers system, and bone repair. HA from cuttlefish bone produces the same effect as HA from bovine bone so that cuttlefish bone-derived HA can be used as an alternative bone filler aside from bovine bone for bone regeneration due to fractures.

## Authors’ Contributions

AA developed the concepts and designed the experiments, evaluated the hydroxyapatite, and wrote the manuscript. FDEH conducted an *in vivo* experiment on the rats. PW conducted the tests and the anatomical pathology analysis as well as wrote the manuscript; DW conducted tests and the anatomical pathology analysis, while SS conducted the ANOVA statistical tests. All authors read and approved the final manuscript.
